# Monitoring mitochondrial function in peripheral T cells to assess immune status and graft health after kidney transplantation

**DOI:** 10.3389/fimmu.2025.1721097

**Published:** 2026-01-13

**Authors:** Erdi Zhang, Zhengli Wan, Yuwen Ma, Yamei Li, Qixiang Zhou, Bei Cai, Yi Li, Feng Li, Binwu Ying, Lin Yan

**Affiliations:** 1 Department of Laboratory Medicine, West China Hospital, Sichuan University, Chengdu, China; 2 Clinical Laboratory Medicine Research Center, West China Hospital, Sichuan University, Chengdu, China; 3 Sichuan Clinical Research Center for Laboratory Medicine, Chengdu, China; 4 Institution of Medical and Engineering Integration for Molecular Diagnosis, West China Hospital, Sichuan University, Chengdu, China; 5 Key Laboratory of Green Chemistry and Technology of Ministry of Education, College of Chemistry, Sichuan University, Chengdu, China

**Keywords:** graft dysfunction, immune monitoring, kidney transplantation, mitochondrial mass, mitochondrial membrane potential, T lymphocytes

## Abstract

**Introduction:**

Kidney transplantation is the preferred treatment for end-stage renal disease, but immune-mediated graft dysfunction remains a major barrier to long-term success. Conventional indicators such as serum creatinine and proteinuria are not immune-specific, and kidney biopsy is unsuitable for routine use. Mitochondrial function, a critical regulator of immune activation, may provide novel biomarkers for immune monitoring in transplantation.

**Methods:**

In this study, the percentage of peripheral immune cells with low mitochondrial membrane potential (MMP-Low%) and mitochondrial mass (MM) were assessed in 30 kidney transplant recipients and 44 healthy controls. Of the 30 transplant recipients, 15 provided paired samples before transplantation and at one month after transplantation with preserved graft function, while the other 15 had impaired graft function. Flow cytometry was used to measure lymphocyte subsets and mitochondrial parameters, which were further evaluated across specific T cell populations.

**Results:**

The results showed that increased age correlated with lower MMP-Low% and higher MM. Patients with impaired graft function showed significantly reduced MMP-Low% and elevated MM, suggesting enhanced metabolic activation, while those with preserved graft function displayed increased MMP-Low% and decreased MM, reflecting a quiescent metabolic profile consistent with immunosuppression.

**Discussion:**

These findings indicate that peripheral T cell mitochondrial metrics may serve as sensitive, dynamic, and non-invasive biomarkers for post-transplant immune monitoring.

## 1 Introduction

Chronic kidney disease (CKD) represents a major global health concern, with many patients ultimately progressing to end-stage renal disease (ESRD) and requiring renal replacement therapy (1, 2). Kidney transplantation offers the best clinical outcomes for these patients, yet long-term graft survival continues to be limited by immune-mediated injury (3–5).

Currently, post-transplant monitoring of immune status and graft function relies primarily on serum creatinine levels and proteinuria, both of which reflect renal function but provide limited information regarding immune activity. As a result, these conventional markers often fail to detect early or subclinical immune activation that precedes overt graft damage. When abnormalities arise, clinicians may resort to renal biopsy, which—despite being the gold standard for distinguishing different pathological types of rejection—is invasive, time-consuming, and not suitable for routine surveillance (6–10). Conventional immune cell counts, such as T, B, and NK lymphocyte enumeration, provide only limited functional insight and often fail to predict subclinical rejection or evolving immune activation. Therefore, there is a pressing need for novel, non-invasive biomarkers that can dynamically capture both immune activation and early graft injury. If validated, such biomarkers could be incorporated into post-transplant surveillance to complement conventional functional indices and provide more immune-specific information. By signaling emerging immune activation before overt dysfunction, they may assist in risk stratification and help guide the timing of biopsy or adjustment of immunosuppressive therapy.

T cells are central mediators of allograft rejection, participating in both acute cellular and chronic antibody-mediated responses (11). Their activation state, differentiation trajectory and metabolic programming are tightly regulated by mitochondrial function, which governs key processes such as energy metabolism, redox balance, apoptosis and cytokine production in T lymphocytes (12, 13). Among mitochondrial parameters, mitochondrial membrane potential (MMP) and mitochondrial mass (MM) are widely used as integrative readouts of immune cell metabolic state and functional integrity. High MMP and increased MM are characteristic of activated effector and memory T cells and correlate with enhanced proliferation, cytokine secretion and cytotoxicity, whereas quiescent or naïve T cells display lower mitochondrial activity and biomass (12, 14). In contrast, chronically stimulated or exhausted T cells, including tumour-infiltrating and terminally exhausted CD8^+^ subsets, accumulate structurally defective, depolarized mitochondria and exhibit reduced mitochondrial mass and membrane potential, which are tightly linked to impaired effector function and an inhibitory or exhausted phenotype (15, 16). Together with data from autoimmune and chronic inflammatory diseases showing mitochondrial dysfunction in T cells (12), these observations support MMP and MM as mechanistic markers that distinguish activated versus suppressive or exhausted immune states. However, although mitochondrial metrics have been explored in autoimmunity, chronic infection and cancer (17–19), to date there has been a lack of studies evaluating their use as non-invasive biomarkers of alloimmune activation and graft injury in the setting of kidney transplantation.

In this study, we aimed to characterize mitochondrial functional parameters of peripheral blood lymphocyte subsets across different clinical stages after kidney transplantation and to explore their relationship with immune activation and graft dysfunction. Specifically, we focused on mitochondrial membrane potential and mitochondrial mass in T, B and NK cells, with particular attention to T-cell subpopulations that may undergo immunometabolic remodeling in response to alloimmune stimulation. We hypothesized that mitochondrial functional parameters of peripheral T cells would correlate with immune activation states and graft outcomes and could serve as minimally invasive biomarkers for post-transplant immune monitoring.

## 2 Materials and methods

### 2.1 Study design and participants

Our cross-sectional study was conducted at West China Hospital, Sichuan University, from September 2023 to April 2024. Data collection and laboratory analyses were performed within the same period. A total of 74 participants contributed 89 peripheral blood samples, divided into four groups: (1) patients with end-stage renal disease prior to transplantation (ESRD, n=15); (2) the same patients re-evaluated at one month post-transplant with preserved graft function, defined as estimated glomerular filtration rate (eGFR) > 60 mL/min/1.73 m² (PGF, n=15); (3) transplant recipients with impaired graft function, defined as eGFR ≤ 60 mL/min/1.73 m² (IGF, n=15); and (4) healthy individuals undergoing routine physical examinations (HC, n=44). Inclusion criteria were adult kidney transplant recipients with available clinical data and peripheral blood samples; exclusion criteria included multi-organ transplantation or infection at sampling. All participants provided informed consent prior to sample collection. Sample size was determined by the availability of eligible participants during the study period, as no prior data were available for power estimation. The study protocol was reviewed and approved by the ethics committee of West China Hospital (Approval No. 2021(218)).

### 2.2 Sample collection and flow cytometry

#### 2.2.1 Peripheral blood TBNK phenotyping and assessment of mitochondrial function

A total of 4 mL of peripheral blood was collected and gently mixed. An aliquot of 100 μL was incubated with 20 μL of a TBNK antibody cocktail (CD8 FITC, CD19 FITC, CD3 PE, CD56 PE, CD45 PerCP^-^Cy5.5, CD4 PE^-^Cyanine7) for 15 min at room temperature in the dark. Then, 2 mL of hemolysin diluted 1:1 with distilled water was added to lyse red blood cells, followed by a further 15 min incubation at room temperature in the dark. After centrifugation at 700 g for 5 min, the supernatant was discarded and the cell pellet was resuspended in 180 μL PBS. A 200^-^μL aliquot was then incubated with the mitochondrial probe MitoDye (C_34_H_36_Cl_2_N_2_; patent no. CN202110570964; UBBiotech (Zhejiang) Co., LTD., Ningbo, China) for 30 min at 37°C in the dark. Finally, the samples were transferred into absolute counting tubes and acquired on a BD FACSCanto™ flow cytometer (BD Biosciences, San Jose, CA, USA).

#### 2.2.2 Mitochondrial function in peripheral blood Tcell functional subsets (TFUN)

An aliquot of 100 μL peripheral blood was incubated with a TFUN antibody mixture containing 20 μL of a multicolor TFUN antibody cocktail and 5 μL each of CD28 and PD^-^1 antibodies (CD4 FITC, CD45RA PerCP^-^Cy5.5, CD62L PE^-^Cyanine7, CD8 APC^-^Cyanine7, CD28 mF540, PD^-^1 PE) for 15 min at room temperature in the dark. Red blood cells were lysed and the samples washed by centrifugation as described in section 2.2.1. The cell pellet was then resuspended in 180 μL PBS, and 200 μL was incubated with MitoDye for 30 min at 37°C in the dark before transfer into absolute counting tubes for flow cytometric analysis.

### 2.3 Flow cytometric gating strategy and analysis

Flow cytometric data were analyzed and gated using NovoExpress software (version 1.4.1; Agilent Technologies, San Diego, CA, USA). Gating strategies for the TBNK and TFUN panels are shown in **Supplementary Figures S1**, **S2**. Lymphocytes were first identified by gating on CD45 versus side scatter (SSC). Major lymphocyte subsets were then distinguished using CD3/CD56 and CD8/CD19 bivariate plots: T cells were defined as CD3^+^CD56^-^, B cells as CD3^-^CD56^-^CD19^+^, and NK cells as CD3^-^CD56^+^. Within the T^-^cell compartment, CD4^+^ T cells were defined as CD4^+^CD8^-^ and CD8^+^ T cells as CD4^-^CD8^+^ based on CD4 and CD8 expression. Furthermore, according to CD45RA and CD62L expression, T cells were subdivided into the following functional subsets: naïve T cells (Tn, CD45RA^+^CD62L^+^), central memory T cells (Tcm, CD45RA^-^CD62L^+^), effector memory T cells (Tem, CD45RA^-^CD62L^-^), and effector T cells (Teff, CD45RA^+^CD62L^-^). Mitochondrial mass (MM) was assessed after 30 min incubation with the mitochondrial probe at 37°C in the dark and was detected in the APC channel of the flow cytometer. MM was expressed as the median fluorescence intensity (MFI) of the mitochondrial probe signal (20–23). Mitochondrial membrane potential–low percentage (MMP^-^Low%) was determined by resolving the fluorescence intensity distribution into negative and positive populations after probe staining; a gate was set on the low^-^fluorescence (negative) population, designated as MMP^-^Low, and MMP^-^Low% was defined as the proportion of MMP^-^Low cells within the target cell population. Raw flow cytometry data files (.fcs) were processed and corrected using a dedicated human lymphocyte mitochondrial function analysis system (software; patent no. CN202210495602.3), and the corrected data were then exported for downstream analysis.

### 2.4 LASSO regression for renal function classification

To identify predictors of renal function status after kidney transplantation, we applied a LASSO-regularized logistic regression model to 55 continuous immune-metabolic variables, including cytokines, inflammation indices, TBNK/TFUN subsets, and mitochondrial markers. The binary outcome was defined by eGFR: patients with eGFR >60 mL/min/1.73 m² were coded as 0 (preserved function), and those with eGFR ≤60 as 1 (impaired function). Modeling was performed using the glmnet package in R, with L1 regularization (alpha=1) and leave-one-out cross-validation (nfolds=30). The optimal λ was selected based on cross-validated AUC. Model performance was evaluated using ROC analysis. Variables with non-zero coefficients at the optimal λ were retained as predictors.

### 2.5 Statistical analysis

All statistical analyses and graphical visualizations were performed using GraphPad Prism (Version 10.1.1, GraphPad Software, USA) and R software (Version 4.4.2, R Foundation for Statistical Computing, Vienna, Austria). For comparisons between groups, pairwise analyses were performed using unpaired t test (parametric) or Mann–Whitney U test (non-parametric), as appropriate. Two-sided P values <0.05 were considered statistically significant. Unless otherwise indicated, data from normally distributed variables are presented as mean ± SD, while data from non-normally distributed variables are presented as median (IQR). One participant in the ESRD group had incomplete measurements for certain variables and was excluded from those analyses; complete-case analysis was applied without data imputation. No sensitivity analyses were performed.

## 3 Results

### 3.1 Participant characteristics

A total of 74 individuals were enrolled, contributing 89 samples: 15 samples in the ESRD group, 15 in the PGF group, 15 in the IGF group, and 44 in HC group. To minimize the confounding effect of age, 16 healthy controls, whose age and gender distributions showed no significant differences compared with the three patient groups, were selected as a matched subgroup for subsequent analyses. Baseline characteristics are summarized in **Table 1**. As expected, eGFR values varied significantly among clinical groups (P < 0.0001), with the lowest levels observed in the ESRD group. Urine protein levels, semi-quantified by dipstick, were markedly elevated in the IGF group compared to the PGF group (P < 0.001).

**Table 1:** Baseline clinical characteristics of study participants across clinical groups.

Clinical Characteristics	ESRD (N=15)	PGF (N=15)	IGF (N=15)	HC (N=16)	P value	HC-all (N=44)
age (year)	34.07 ± 9.22	34.07 ± 9.22	37.27 ± 9.79	37.07 ± 4.58	0.5026	55.07 ± 15.24
gender (Male/Female)	8/7	8/7	12/3	9/7	0.3682	22/22
eGFR (mL/min/1.73m²)	4.49 ± 1.87	73.39 ± 14.51	38.23 ± 17.44	/	<0.0001	/
Urine protein						
−	/	8	1	/	<0.001	/
±	/	2	1	/		/
+	/	3	2	/		/
++	/	2	5	/		/
+++	/	0	5	/		/
++++	/	0	1	/		/

### 3.2 Age-associated changes in mitochondrial function of lymphocyte subsets

**Figure 1:**
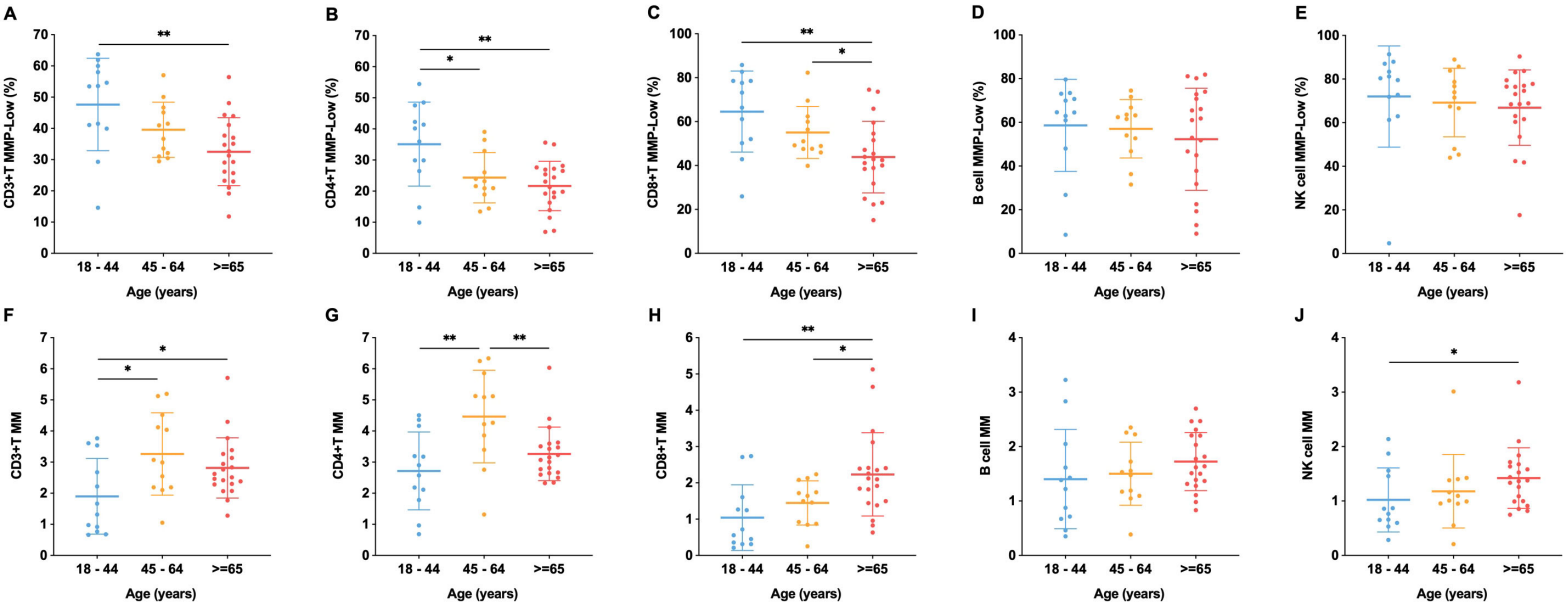
Age-associated alterations in mitochondrial membrane potential and mass across lymphocyte subsets. Mitochondrial membrane potential [MMP-Low%, **(A, E)**] and mitochondrial mass [MM, **F–J**] were measured by flow cytometry in CD3^+^ T cells **(A, F)**, CD4^+^ T cells **(B, G)**, CD8^+^ T cells **(C, H)**, B cells **(D, I)**, and NK cells **(E, J)** across three age groups: 18–44 years, 45–64 years, and ≥65 years. *P < 0.05, **P < 0.01.

To establish the physiological relevance of mitochondrial indicators, we first examined their association with age in the entire cohort of HC (N=44), given the known impact of immunosenescence on immune cell metabolism and mitochondrial dynamics (24–26). Among healthy individuals, aging was associated with a decline in MMP-Low% and an increase in MM across T, B, and NK cell populations. Specifically, CD3^+^, CD4^+^, and CD8^+^ T cells showed significantly reduced MMP-Low% in the ≥he-Low% subgroup compared to those aged 18–44 years (P < 0.01), and all lymphocyte subsets displayed a general trend of declining MMP-Low% with increasing age. With respect to mitochondrial mass, age-related increases were most pronounced in cytotoxic populations, including CD8^+^ T cells and NK cells, whereas helper CD4^+^ T cells exhibited the most prominent elevation in the 45evationn subgroup (**Figures 1A–J**). Because this analysis demonstrated that age significantly influences MMP-Low% and MM, we subsequently selected an age-matched HC subgroup (N=16) for comparisons with patient groups in order to minimize age-related confounding. In addition, we examined the effect of sex on mitochondrial parameters within the healthy population and found no significant differences between males and females (**Supplementary Figure S3**).

### 3.3 Altered mitochondrial function in different clinical stages of kidney transplantation

**Figure 2:**
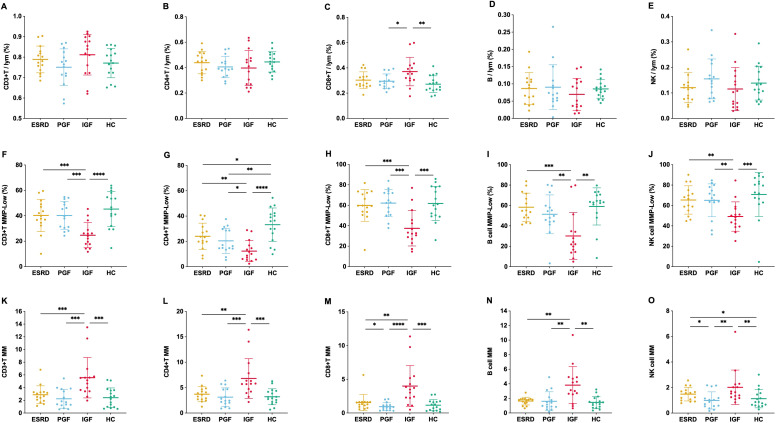
Lymphocyte subset proportions and mitochondrial parameters across clinical groups. Proportions of lymphocyte subsets **(A–E)**, mitochondrial membrane potential [MMP-Low%, **(F–J)**], and mitochondrial mass [MM, **(K–O)**] were measured by flow cytometry in CD3^+^ T cells **(A, F, K)**, CD4^+^ T cells **(B, G, L)**, CD8^+^ T cells **(C, H, M)**, B cells **(D, I, N)**, and NK cells **(E, J, O)** among four groups. *P < 0.05, **P < 0.01, ***P < 0.001, ****P < 0.0001.

To further evaluate immune status after kidney transplantation, we compared lymphocyte subsets and their mitochondrial function across clinical groups. Overall, the proportions of T, B, and NK cells (TBNK) remained generally stable among the four groups, with an increased proportion of CD8^+^ T cells observed only in the IGF group. In contrast, the IGF group exhibited significantly decreased MMP-Low% and increased mitochondrial mass (MM) in CD3^+^, CD4^+^, CD8^+^ T cells, B cells, and NK cells (P < 0.05). Meanwhile, no marked differences in MMP-Low% or MM were observed between the ESRD and PGF groups across the major lymphocyte subsets (**Figures 2A–O**). These results indicate that mitochondrial function remains largely stable before and after transplantation but shows pronounced abnormalities in the presence of graft dysfunction, reflecting a hypermetabolic or stress-activated lymphocyte state.

### 3.4 Mitochondrial remodeling in T cell subsets

Given that T cells exhibited the most robust and widespread mitochondrial differences among lymphocyte subsets, we further analyzed their functional compartments. The alterations in the IGF group extended to CD4^+^ and CD8^+^ Tn, Tcm, Teff, Tem, and CD28^+^ subsets.

**Figure 3:**
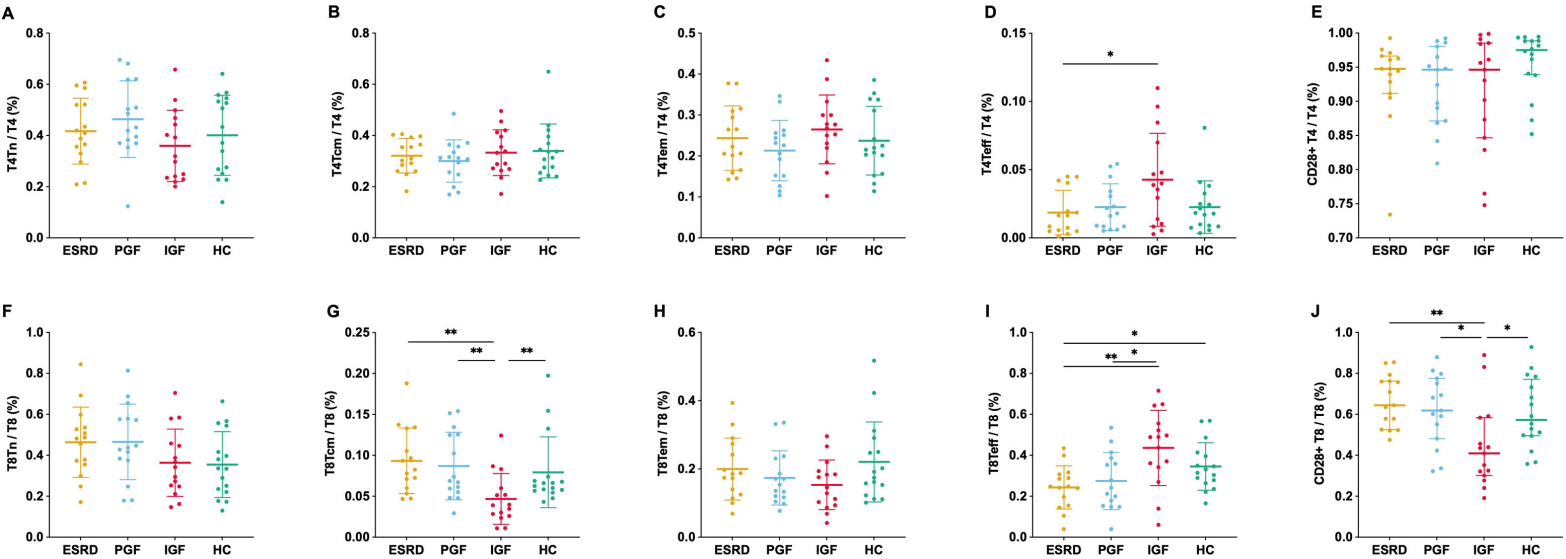
Proportions of T cell subsets in different clinical groups. The relative proportions of CD4^+^ and CD8^+^ T cell subsets were measured by flow cytometry, including naïve [T4Tn, **(A)**; T8Tn, **(F)**], central memory [T4Tcm, **(B)**; T8Tcm, **(G)**], effector memory [T4Tem, **(C)**; T8Tem, **(H)**], effector [T4Teff, **(D)**; T8Teff, **(I)**], and CD28^+^ subsets [CD28^+^ T4, **(E)**; CD28^+^ T8, **(J)**] across four groups. *P < 0.05, **P < 0.01.

The overall distribution of CD4^+^ and CD8^+^ subsets remained relatively stable across the four groups, with significant differences observed only in specific subsets. Specifically, T4Teff was significantly increased in IGF compared with ESRD, whereas T8Tcm was significantly decreased. For CD28^+^ subsets, HC showed higher proportions in T4Tcm and T8Tcm, while IGF showed markedly lower proportions in total T8 and T8Tcm (**Figure 3**).

**Figure 4:**
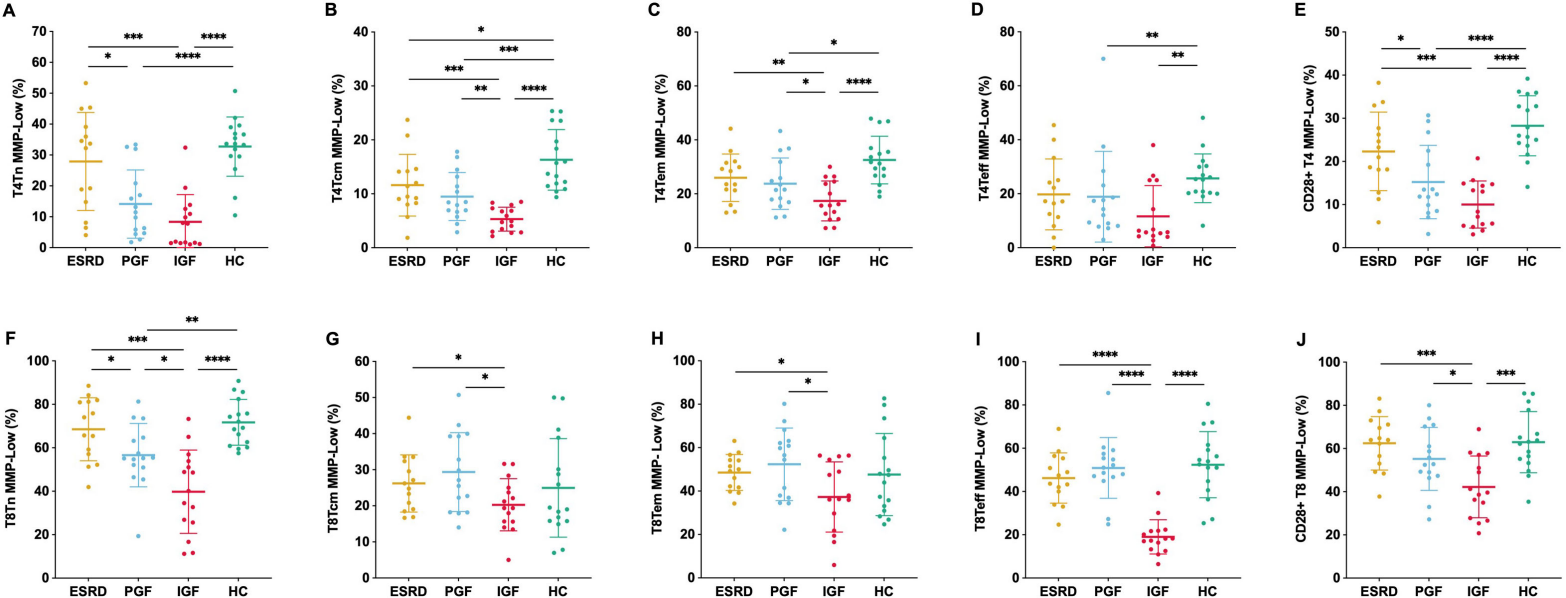
Mitochondrial membrane potential (MMP-Low%) across T cell subsets in different clinical groups. MMP-Low% was assessed in CD4^+^ T cell subsets—naïve [T4Tn, **(A)**, central memory [T4Tcm, **(B)**], effector memory [T4Tem, **(C)**, effector [T4Teff, **(D)**, and CD28^+^**(E)**]—and CD8^+^ T cell subsets—naïve [T8Tn, **(F)**, central memory [T8Tcm, **(G)**, effector memory [T8Tem, **(H)**, effector [T8Teff, **(I)**, and CD28^+^**(J)**]—across four groups. *P < 0.05, **P < 0.01, ***P < 0.001, ****P < 0.0001.

MMP-Low% was significantly reduced in nearly all subsets in IGF compared with the other groups, with the most pronounced differences observed in CD4^+^ Tn, Tcm, Tem, CD28^+^ T cells as well as CD8^+^ Tn, Teff, and CD28^+^ T cells. In CD4^+^ Tn, Tcm, Tem, Teff, and CD28^+^ subsets, MMP-Low% was lower in ESRD than in HC, decreased further in PGF, and reached the lowest levels in IGF, showing a stepwise decline. A similar progressive reduction across the groups was also observed in CD8^+^ Tn and CD28^+^ subsets (**Figure 4**).

**Figure 5:**
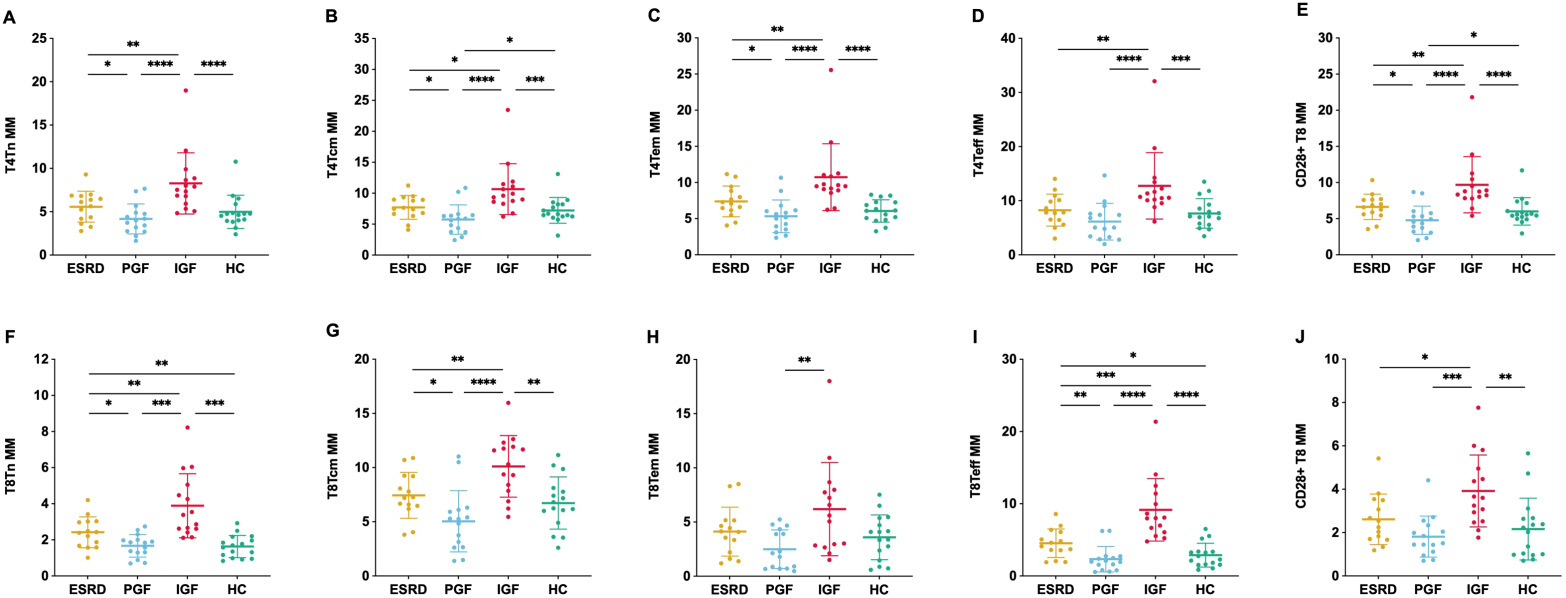
Mitochondrial mass (MM) across T cell subsets in different clinical groups. MM was assessed in CD4^+^ T cell subsets—naïve [T4Tn, **(A)**, central memory [T4Tcm, **(B)**], effector memory [T4Tem, **(C)**, effector [T4Teff, **(D)**, and CD28^+^**(E)]**—and CD8^+^ T cell subsets—naïve [T8Tn, **(F)**, central memory [T8Tcm, **(G)**, effector memory [T8Tem, **(H)**, effector [T8Teff, **(I)**, and CD28^+^**(J)**]—across four groups. *P < 0.05, **P < 0.01, ***P < 0.001, ****P < 0.0001.

MM in most subsets showed no significant differences between ESRD and HC, while PGF was generally lower than ESRD. In contrast, MM in IGF was consistently higher across all subsets. Overall, MM showed a trend of decreasing from ESRD to PGF, followed by an increase in IGF (**Figure 5**).

### 3.5 Systemic inflammatory signatures in IGF group

**Figure 6:**
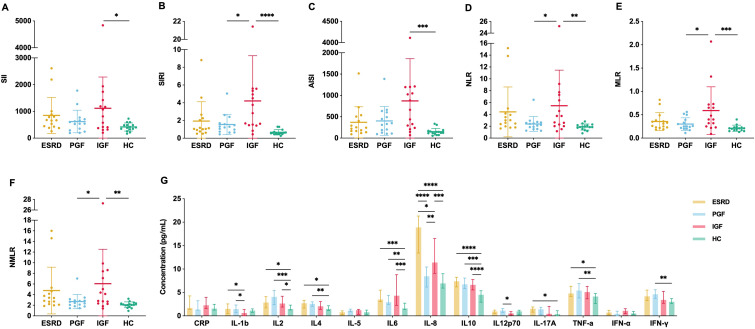
Inflammatory indices and cytokine profiles in peripheral blood across clinical groups. **(A–F)** Systemic inflammatory markers derived from complete blood counts: systemic immune–inflammation index (SII), systemic inflammation response index (SIRI), aggregate index of systemic inflammation (AISI), neutrophil-to-lymphocyte ratio (NLR), monocyte-to-lymphocyte ratio (MLR), and neutrophil–monocyte–lymphocyte ratio (NMLR) were compared across four groups. **(G)** Plasma concentrations of multiple cytokines measured across four groups. *P < 0.05, **P < 0.01, ***P < 0.001, ****P < 0.0001.

To compare the ability of inflammatory indices, cytokines, and mitochondrial parameters to reflect the immune process before and after transplantation, we analyzed routine blood-derived inflammatory indices and circulating cytokine levels. Compared with HC, the IGF group showed significant increases in all six inflammatory indices (SII, SIRI, AISI, NLR, MLR, NMLR) (**Figures 6A-F**); compared with PGF, IGF also had higher levels of SIRI, NLR, MLR, and NMLR.

For cytokines, plasma levels of IL-1b, IL-2, IL-4, IL-6, and IL-10 were generally elevated in transplant recipients compared with HC (**Figure 6G**). However, considerable overlap was observed among ESRD, PGF, and IGF, and no consistent patterns were found across different cytokines. In contrast, IL-8 exhibited a trajectory consistent with MM: higher in ESRD, decreased in PGF, and increased again in IGF. Overall, although inflammatory indices and cytokines can reflect the proinflammatory milieu in IGF, most markersammator IL-8ersam limited ability to capture the immune process before and after transplantation. By comparison, mitochondrial parameters more sensitively and continuously delineated the changes from ESRD to PGF and then to IGF.

### 3.6 LASSO identifies CD8^+^ Teff (T8Teff) MMP-Low% as a key post-transplant predictor of graft function

**Figure 7:**
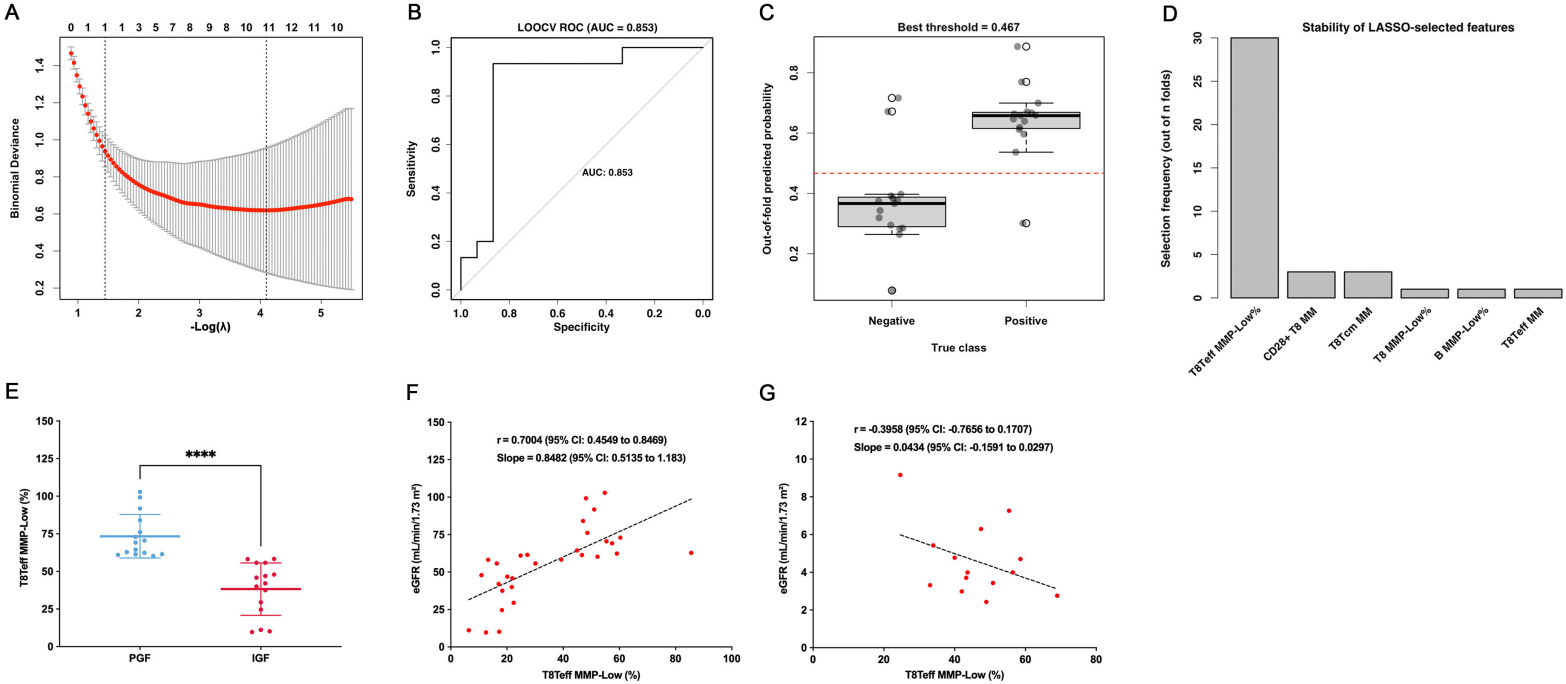
LASSO logistic regression identifies T8Teff MMP-Low% as the key predictor of graft function. **(A)** Cross-validation curve of binomial deviance across λ values. **(B)** ROC curve of leave-one-out cross-validation (LOOCV). **(C)** Out-of-fold predicted probabilities for each true class. **(D)** Feature stability analysis showing that only T8Teff MMP-Low% was consistently retained among 55 input variables. **(E)** Group comparison of T8Teff MMP-Low% between PGF and IGF groups (unpaired t test). **(F)** Correlation and linear regression analysis of T8Teff MMP-Low% with eGFR in the post-transplant setting (PGF and IGF groups). **(G)** Correlation and linear regression analysis of T8Teff MMP-Low% with eGFR in the pre-transplant ESRD group.

LASSO-regularized logistic regression was applied to identify immune-metabolic predictors of graft dysfunction in the post-transplant setting. A total of 55 candidate variables were initially included in the model. The optimal λ was determined by minimizing binomial deviance during cross-validation (**Figure 7A**). Leave-one-out cross-validation (LOOCV) demonstrated robust discriminative performance with an AUC of 0.853 (**Figure 7B**). Out-of-fold predicted probabilities showed clear separation between PGF and IGF (**Figure 7C**). Notably, among all 55 variables entered, only T8Teff MMP-Low% was consistently retained across folds, underscoring its stability as the key predictor (**Figure 7D**). Group comparison further revealed that T8Teff MMP-Low% was significantly higher in PGF group compared with IGF group (P < 0.0001, unpaired t test; **Figure 7E**). Moreover, correlation and linear regression analysis demonstrated a strong positive association between T8Teff MMP-Low% and eGFR in the post-transplant setting (PGF and IGF groups; r=0.7004, P < 0.0001; slope=0.8492; **Figure 7F**). By contrast, no significant correlation was observed in the pre-transplant ESRD group (r=−0.3958, P=0.1613; slope=0.0434; **Figure 7G**).

## 4 Discussion

Effective immune monitoring after kidney transplantation remains a major clinical challenge. Conventional biomarkers such as serum creatinine and proteinuria lack sensitivity and immunological specificity, while standard lymphocyte subset quantification reflects only numerical changes without capturing functional states. Given the central role of mitochondria in regulating immune metabolism and effector activity, mitochondrial profiling may offer an opportunity to resolve immune alterations that are otherwise missed by conventional assays.

In ESRD and PGF patients, we observed a shared overall trend toward higher MMP-Low% and lower MM across TBNK and TFUN subsets. This partially overlapping mitochondrial pattern suggests that reduced mitochondrial engagement may be a common feature of immunosuppressed immune states, arising from uremia-associated immune dysfunction in ESRD or from pharmacological immunosuppression after transplantation. In ESRD, chronic exposure to uremic toxins and low-grade inflammation has been linked to impaired mitochondrial function, increased oxidative stress and an accelerated immunosenescent phenotype, thereby constraining the metabolic reserve and functional responsiveness of immune cells (27, 28). The PGF-associated pattern can be further understood in the context of the known mitochondrial effects of standard maintenance immunosuppressive agents. Sirolimus, for instance, has been shown to significantly reduce mitochondrial respiration in thymic lymphocytes, providing direct evidence that mTOR inhibition dampens immunometabolic activity in immune compartments (29, 30). Mechanistic studies further indicate that mTOR blockade downregulates mitochondrial biogenesis and translation programs via suppression of YY1/PGC-1o and 4E-BPGC-1onalTE signaling, thereby limiting oxidative phosphorylation and metabolic fitness (31, 32). Calcineurin inhibition has been linked to pyruvate dehydrogenase deactivation and impaired mitochondrial energy metabolism, reducing the ability of T cells to sustain effector functions (33). In addition, IMPDH inhibition by mycophenolate depletes the guanine nucleotide pool, constraining GTP-dependent mitochondrial processes such as mtDNA replication, translation and fission dynamics (34, 35). Collectively, these findings support the interpretation that this shared reduction in mitochondrial activation in ESRD and PGF reflects a metabolically constrained, immunosuppressed immune milieu shaped by uremia- or drug-induced inhibition of immunometabolic pathways, depending on the clinical setting, rather than representing a naturally quiescent baseline state.

By contrast, recipients with IGF showed widespread mitochondrial remodeling across T, B and NK cells as well as TFUN subsets, with lower MMP-Low% accompanied by higher MM. Independent studies have described related mitochondrial changes in the transplant setting. For example, one analysis integrating kidney-transplant datasets identified mitophagy- and mitochondria-related gene signatures and confirmed their upregulation by qPCR in PBMCs from recipients with acute rejection, suggesting that mitochondrial programs in circulating immune cells are mobilized during graft injury (36). Mitophagy-related gene signatures have also been used to predict delayed graft function and long-term allograft loss after kidney transplantation, further linking altered mitochondrial quality control to clinically relevant outcomes (37). In addition, modulation of mitophagy pathways, such as PINK1–Parkin- or AMBRA1-dependent mitophagy, has been reported to influence graft inflammation, fibrosis and rejection in experimental transplant settings (38, 39). These observations are consistent with the notion that mitochondrial mass and membrane potential are shaped not only by respiratory demand but also by dynamic quality-control processes: biogenesis driven by PGC-1α/NRF/TFAM increases mitochondrial copy number and cristae complexity, fusion–fission cycles sculpt mitochondrial networks, and mitophagy selectively removes depolarized, ROS-producing organelles (40–42). Thus, one plausible explanation is that the IGF profile of reduced MMP-Low% with increased MM may reflect enhanced mitochondrial engagement together with active remodeling of mitochondrial dynamics and quality-control programs in the setting of allograft injury.

In addition to mitochondrial remodeling, systemic inflammation indices (SII, SIRI, AISI, NLR, MLR, NMLR) and cytokines such as IL-2, IL-6, IL-8 and IL-10 were elevated in the IGF group, indicating a pro-inflammatory milieu. However, with the exception of IL-8, most inflammatory markers showed considerable overlap among ESRD, PGF and IGF and did not reproduce the clear stage-related gradients observed for mitochondrial parameters. A plausible explanation is that the two types of readouts capture different layers of the immune response. Mitochondrial metrics are measured directly in defined lymphocyte subsets and reflect relatively slow, cell-intrinsic metabolic adaptations to cumulative immune activation and pharmacological pressure (13). By contrast, circulating cell-count. CITE indices and soluble cytokines have short half-lives, fluctuate rapidly, and are strongly influenced by non-alloimmune factors such as intercurrent infection, surgical stress, dialysis exposure and comorbid inflammation (43, 44). In a cross-sectional cohort with modest sample size, these sources of biological and technical variability are likely to blur group differences for inflammatory indices, whereas mitochondrial parameters may appear more robust because they integrate immune or treatment history over a longer time window.

Within this context, IL-8 emerged as a notable exception among inflammatory markers and therefore warranted separate consideration. In our cohort, IL-8 displayed a clear gradient across clinical groups, with the highest levels in ESRD, partial reduction after transplantation in PGF, and persistently elevated concentrations in IGF compared with healthy controls. Immunologically, IL-8 (CXCL8) is not only a potent neutrophil chemoattractant but also a canonical component of the senescence-associated secretory phenotype, and sustained IL-8 production has been linked to inflammaging and immunosenescence in chronic disease states (45, 46). In CKD, elevated IL-8 is thought to reflect a uremia-driven inflammatory and vasculopathic milieu (47) rather than rejection-specific activation, whereas experimental data indicate that mitochondrial dysfunction and excessive mitochondrial ROS can further amplify IL-8 expression through NF-κB–dependent pathways (48). Within this framework, IL-8 may therefore capture features of both CKD-associated systemic inflammaging and graft-related immune activation, providing information that complements, rather than simply overlaps with, mitochondrial metrics.

Of particular interest, we identified T8Teff MMP-Low% as the only variable retained in a LASSO-regularized logistic regression model for distinguishing patients with preserved versus impaired renal function. This marker demonstrated strong discriminative power (AUC=0.853) despite the high dimensionality of the input panel. Biologically, elevated MMP-Low% in CD8^+^ Teff cells may reflect reduced mitochondrial activation and a non-inflammatory or immunosuppressed phenotype, consistent with prior studies showing that quiescent or tolerized T cells exhibit diminished mitochondrial respiration and membrane polarization (49). In contrast, activated CD8^+^ T cells undergoing effector differentiation typically exhibit increased mitochondrial potential, ROS production, and metabolic flux via oxidative phosphorylation (13, 50). Thus, patients with preserved renal function in our cohort may display a predominance of metabolically quiescent CD8^+^ Teff cells, potentially reflecting effective immunosuppression or immune accommodation. Importantly, in the ESRD (pre-transplant) cohort, T8Teff MMP-Low% did not correlate with eGFR, likely because eGFR at that stage is dominated by irreversible structural loss (nephron dropout, fibrosis, vascular rarefaction) (51, 52) rather than by dynamic immuno-metabolic activity.

Taken together, mitochondrial dysfunction and remodeling are increasingly recognized as critical regulators of immune cell function (53–58). In T cells, MMP reflects mitochondrial health and coupling efficiency; its loss may signal depolarization or early exhaustion (59), whereas elevated MM can indicate increased metabolic demand or biogenesis (60, 61). Such changes are central to the bioenergetic reprogramming that underlies T-cell differentiation and effector function in inflammation and transplantation. Within the limitations of a small, cross-sectional cohort, our results are consistent with the view that peripheral lymphocyte mitochondrial state may serve as an *in vivo* correlate of immune activation during allograft stress, and they generate testable hypotheses for future longitudinal and mechanistic studies.

The study was limited by a relatively small, cross-sectional cohort, which reduces the strength of causal inference and generalizability. Moreover, the considerable overlap in MMP-Low% and MM between ESRD and PGF suggests that mitochondrial metrics alone may have limited discriminatory power between different immunosuppressed states, particularly when comparing uremia-associated and pharmacologically induced immune suppression. In addition, analyses were confined to peripheral blood lymphocytes, so it remains unclear to what extent the observed mitochondrial alterations mirror immune activity within the graft itself. Accordingly, our findings should be interpreted as observational associations that primarily generate testable hypotheses regarding the relationships between mitochondrial parameters, immune activation and graft outcomes, rather than as definitive mechanistic evidence.

## 5 Conclusions

Mitochondrial profiling of peripheral lymphocytes reveals distinct immunometabolic signatures across different clinical states of kidney transplantation. Patients with PGF showed reduced mitochondrial engagement consistent with pharmacological immunosuppression, whereas IGF was marked by enhanced mitochondrial polarization and biogenesis across T, B, and NK compartments. Among these changes, CD8^+^ Teff MMP-Low% emerged as a parsimonious and functionally meaningful biomarker, demonstrating strong discriminative power for graft function status. These findings suggest that mitochondrial parameters provide added value beyond conventional immune monitoring, offering a sensitive and stable readout of immune activation. Longitudinal studies linking mitochondrial metrics with histopathology and clinical outcomes will be critical to validate their predictive utility and to define their role in guiding personalized immunosuppression strategies.

## Data Availability

The original contributions presented in the study are included in the article/**Supplementary Material**. Further inquiries can be directed to the corresponding author.
