# Supplementary material for: Monitoring mitochondrial function in peripheral T cells to assess immune status and graft health after kidney transplantation
Source: Front Immunol. 2026 Jan 13;16:1721097. doi: 10.3389/fimmu.2025.1721097 (PMC12834765; doi:10.3389/fimmu.2025.1721097)
Supplement: Supplementary file 1 [file DataSheet1.pdf]

Supplementary Material

Supplementary Figures

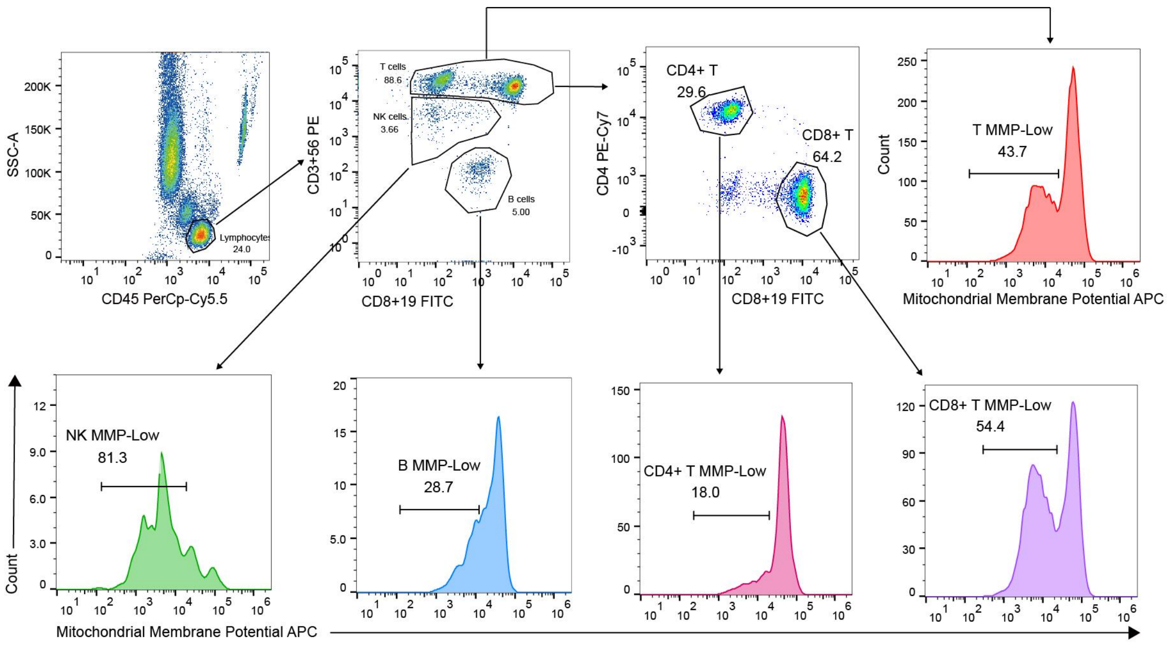

FIGURE S1

Gating strategy for major peripheral blood lymphocyte populations and mitochondrial function analysis. Lymphocytes were classified as $CD4^+$ helper T cells (Th), $CD8^+$ T cells (Ts), B cells (B), and natural killer cells (NK), with granulocytes shown as a reference. MMP-Low% was quantified within each gated population using the mitochondrial probe, and neutrophils with intrinsically low MMP served as an internal reference for gate calibration.

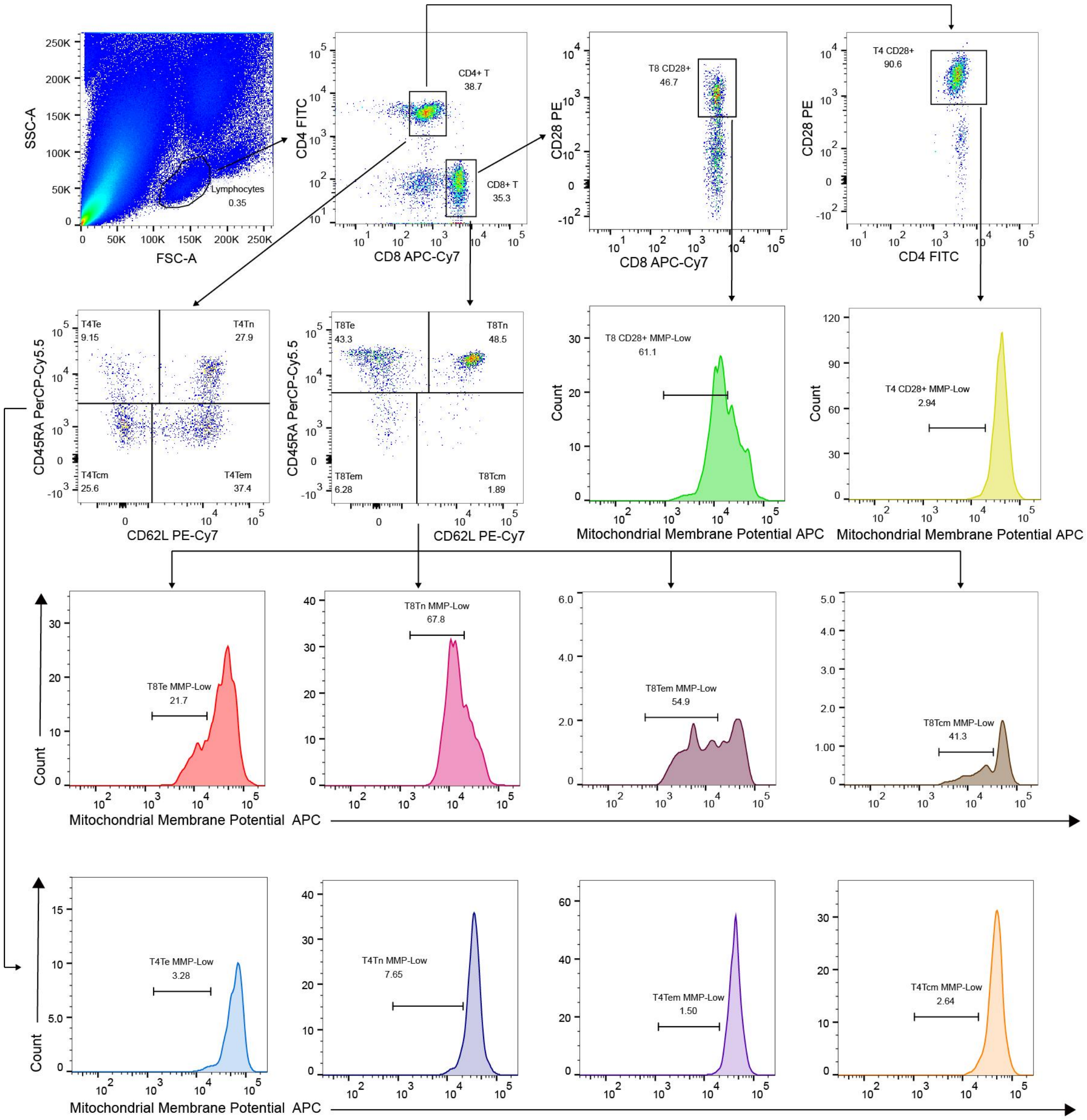

FIGURE S2

Gating strategy for CD4$^+$ and CD8$^+$ T cell subsets and mitochondrial function assessment. Lymphocytes were first identified by FSC/SSC properties, followed by gating on CD4$^+$ or CD8$^+$ T cells. Subsets were further defined as naïve (Tn), central memory (Tcm), effector memory (Tem), and effector (Teff) cells based on CD45RA and CD62L expression, with CD28$^+$ populations separately gated. The proportion of cells with low mitochondrial membrane potential (MMP-Low%) was quantified within each subset using fluorescence intensity distribution of the mitochondrial probe.

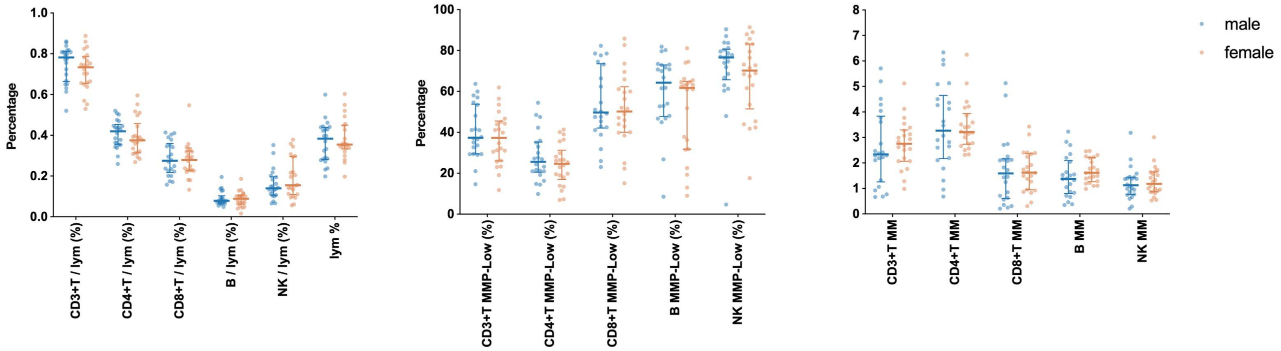

FIGURE S3

Sex-associated differences in lymphocyte subsets and mitochondrial parameters in healthy controls. The proportions of $CD3^+$, $CD4^+$, and $CD8^+$ T cells, B cells, and NK cells (left), their corresponding MMP-Low% values (middle), and mitochondrial mass (MM) (right) are shown for males (blue) and females (orange). Data are presented as mean ± SD or median ± interquartile range (IQR). Intergroup differences were analyzed using unpaired t test or Mann–Whitney U test, as appropriate. A two-sided P value < 0.05 was considered statistically significant. No significant sex-related differences were observed across lymphocyte subsets or mitochondrial indices.
